# Supplementary material for: Structural and Functional Characterization of Stx2k, a New Subtype of Shiga Toxin 2
Source: Microorganisms. 2019 Dec 18;8(1):4. doi: 10.3390/microorganisms8010004 (PMC7022315; doi:10.3390/microorganisms8010004)
Supplement: Supplementary file 1 [file microorganisms-08-00004-s001.zip › Supp material/Supp figs.docx]

**Supporting Figure 1. Amino Acid Sequence alignment of Stxs**. **A)** Sequence alignment of the A-subunit of Stx1a, Stx2a, Stx2e, and Stx2k. Residues conserved in all sequences are shaded in Black, residues with conserved properties are shaded in grey, non-identical resides with dissimilar properties are not shaded. The catalytic residue is indicated by a letter “C” underneath the sequences. The active center residues are indicated by a letter “A” underneath the sequences. **B)** Sequence alignment of the B-subunit of Stx1a, Stx2a, Stx2e, and Stx2k. Shading is the same as above. Residues that constitute binding sites 1, 2, and 3, for the glycosphingolipid receptor globotriaosylceramide and its analogs are indicated by the numbers “1,” “2,” and “3” underneath the sequences, respectively.
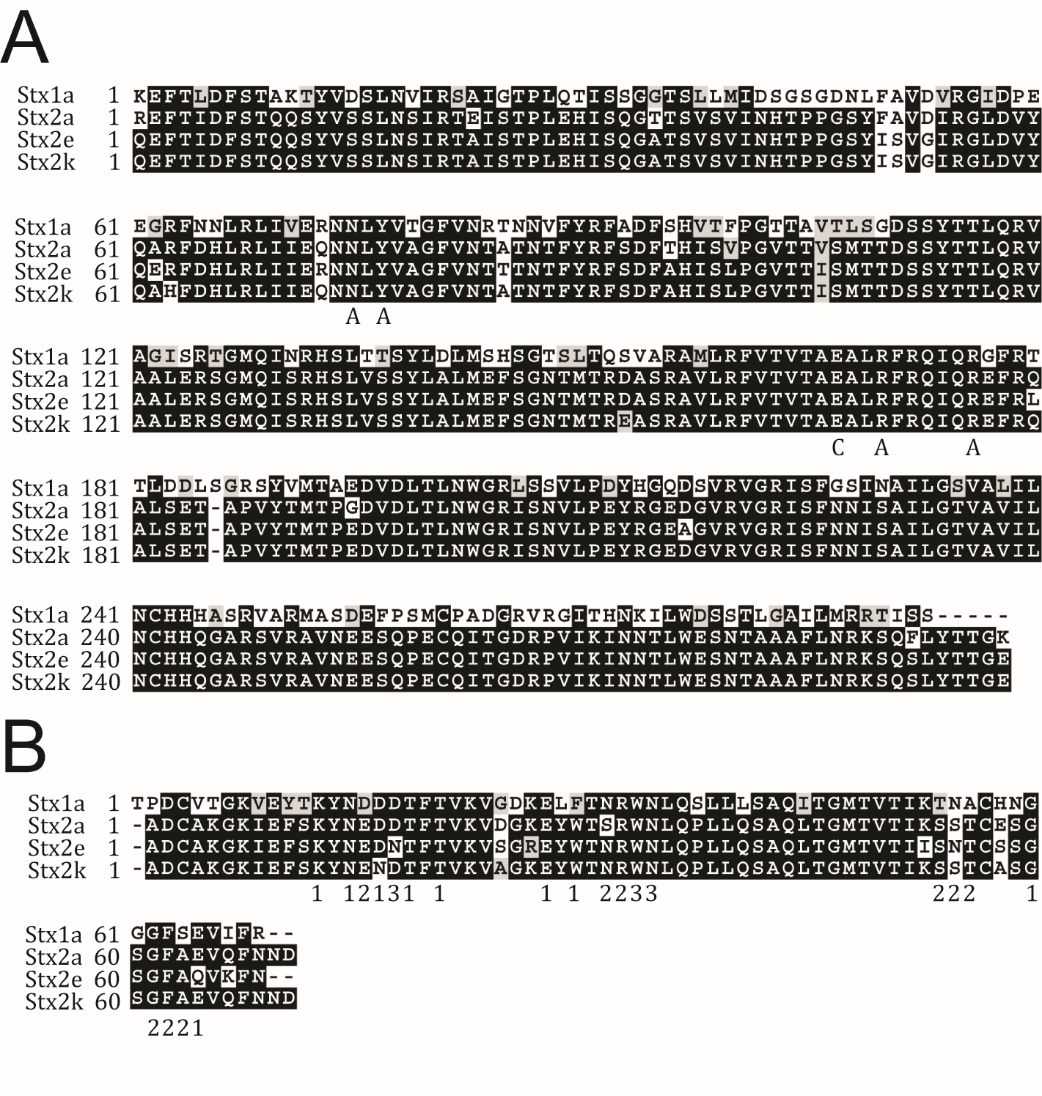


**
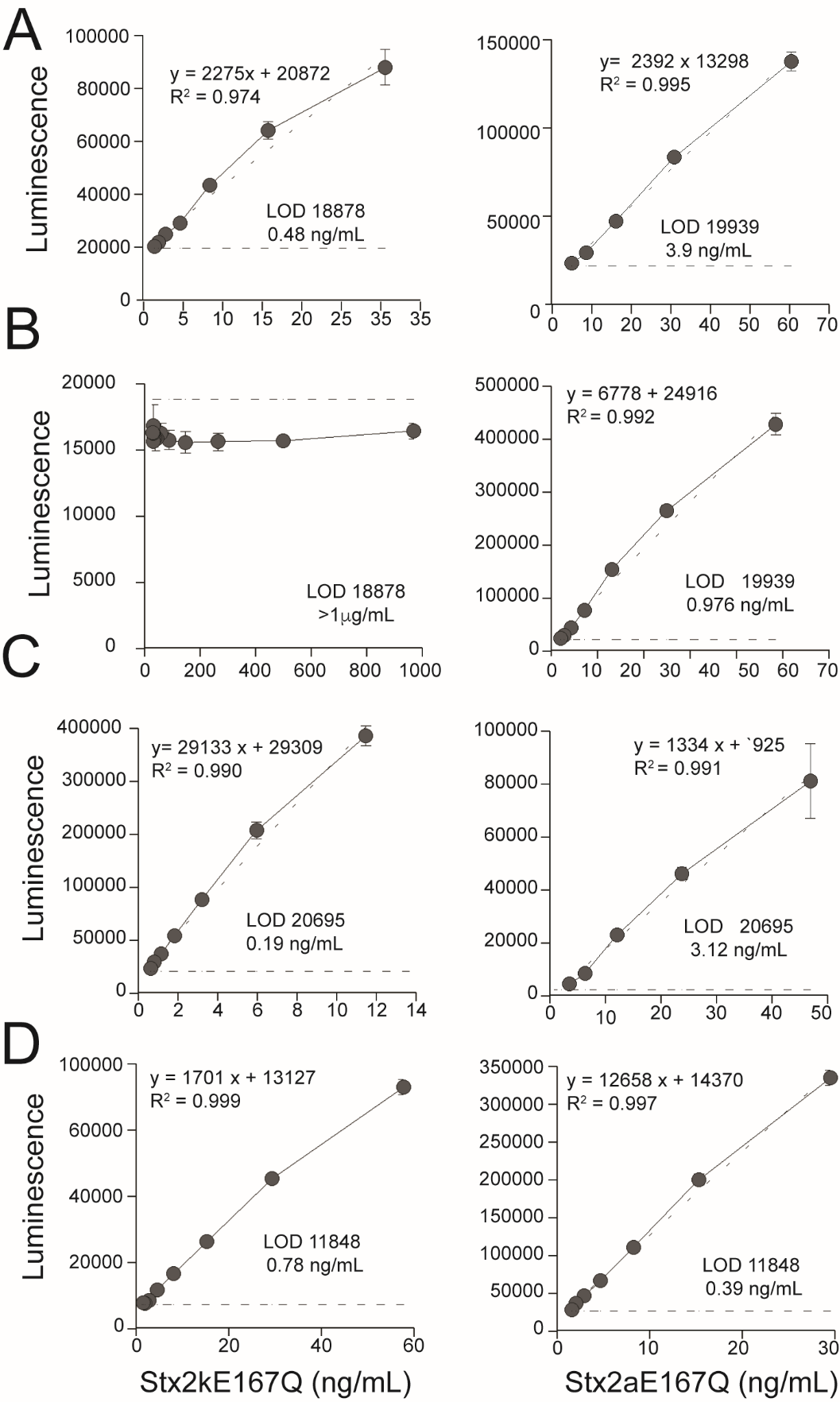
**

**Supporting Figure 2.** Stx2k (Left column) and Stx2a (Right column) standard curves determined by Sandwich ELISAs. Stx2kE167Q and Stx2aE167Q were serially diluted 2-fold from 100ng/mL to 6pg/mL in 1 X PBS and the curves within the linear ranges are shown. 1 X PBS was used as the negative control. Data represent the mean of triplicate counts ± SD. The horizontal dashed line indicates the mean of triplicate luminescence counts from the PBS control plus three SD. The antibody pairs used were **A)** Capturer pAb Stx2a, Detector mAb Stx2e-3, **B)** Capturer pAb Stx2a, Detector mAb Stx2a-2 **C)** Capturer pAb Stx2k, Detector mAb Stx2e-3, and **D)** Capturer mAb Stx2e-3, Detector pAb Stx2a.

**Supplemental Table 1. Data collection and refinement statistics.**

| Wavelength | **0.9793** |
| --- | --- |
| Resolution range | 48.71 - 2.287 (2.369 - 2.287) |
| Space group | P 1 21 1 |
| Unit cell | 57.178 157.022 107.407 90 94.615 90 |
| Total reflections | 164826 (16504) |
| Unique reflections | 83987 (8383) |
| Multiplicity | 2.0 (2.0) |
| Completeness (%) | 98.80 (99.53) |
| Mean I/sigma(I) | 14.49 (2.34) |
| Wilson B-factor | 59.90 |
| R-merge | 0.02382 (0.2796) |
| R-meas | 0.03369 (0.3954) |
| R-pim | 0.02382 (0.2796) |
| CC1/2 | 0.999 (0.871) |
| CC* | 1 (0.965) |
| Reflections used in refinement | 83972 (8383) |
| Reflections used for R-free | 1992 (200) |
| R-work | 0.1971 (0.2897) |
| R-free | 0.2254 (0.3143) |
| CC(work) | 0.964 (0.847) |
| CC(free) | 0.954 (0.747) |
| Number of non-hydrogen atoms | 10054 |
| macromolecules | 9879 |
| ligands | 59 |
| solvent | 116 |
| Protein residues | 1266 |
| RMS(bonds) | 0.005 |
| RMS(angles) | 1.00 |
| Ramachandran favored (%) | 97.09 |
| Ramachandran allowed (%) | 2.83 |
| Ramachandran outliers (%) | 0.08 |
| Rotamer outliers (%) | 0.18 |
| Clash score | 3.93 |
| Average B-factor | 68.81 |
| macromolecules | 68.83 |
| ligands | 85.35 |
| solvent | 58.14 |

Statistics for the highest-resolution shell are shown in parentheses.
